# Association of functional and structural social support with medication adherence among individuals treated for coronary heart disease risk factors: Findings from the REasons for Geographic and Racial Differences in Stroke (REGARDS) study

**DOI:** 10.1371/journal.pone.0198578

**Published:** 2018-06-27

**Authors:** Favel L. Mondesir, April P. Carson, Raegan W. Durant, Marquita W. Lewis, Monika M. Safford, Emily B. Levitan

**Affiliations:** 1 Department of Epidemiology, School of Public Health, University of Alabama at Birmingham, Birmingham, Alabama, United States of America; 2 Department of Medicine, School of Medicine, University of Alabama at Birmingham, Birmingham, Alabama, United States of America; 3 Department of Surgery, Division of Public Health Sciences, Washington University School of Medicine, St. Louis, Missouri, United States of America; 4 Department of Medicine, Weill Cornell Medicine, New York, New York, United States of America; University of Kentucky, UNITED STATES

## Abstract

**Background:**

Functional social support has a stronger association with medical treatment adherence than structural social support in several populations and disease conditions. Using a contemporary U.S. population of adults treated with medications for coronary heart disease (CHD) risk factors, the association between social support and medication adherence was examined.

**Methods:**

We included 17,113 black and white men and women with CHD or CHD risk factors aged ≥45 years recruited 2003–2007 from the REasons for Geographic and Racial Differences in Stroke (REGARDS) study. Participants reported their perceived social support (structural social support: being partnered, number of close friends, number of close relatives, and number of other adults in household; functional social support: having a caregiver in case of sickness or disability; combination of structural and functional social support: number of close friends or relatives seen at least monthly). Medication adherence was assessed using a 4-item scale. Multi-variable adjusted Poisson regression models were used to calculate prevalence ratios (PR) for the association between social support and medication adherence.

**Results:**

Prevalence of medication adherence was 68.9%. Participants who saw >10 close friends or relatives at least monthly had higher prevalence of medication adherence (PR = 1.06; 95% CI: 1.00, 1.11) than those who saw ≤3 per month. Having a caregiver in case of sickness or disability, being partnered, number of close friends, number of close relatives, and number of other adults in household were not associated with medication adherence after adjusting for covariates.

**Conclusions:**

Seeing multiple friends and relatives was associated with better medication adherence among individuals with CHD risk factors. Increasing social support with combined structural and functional components may help support medication adherence.

## Introduction

Medications can reduce the risk of coronary heart disease (CHD) events and mortality among people with known CHD and/or CHD risk factors such as diabetes, hypertension, and dyslipidemia [1–3]. However, a meta-analysis indicated that only 50 to 66% of patients were adherent to cardiovascular medications [4]. Some evidence suggests that social support promotes medication adherence in chronic disease management [5–10]. Social networks provide social support via a series of relationships and interconnectedness through which members influence each other’s behaviors by their daily interactions and feedback mechanisms [11]. These networks may increase treatment adherence through support received from relatives and friends as well as assistance provided for self-care activities [9]. However, social network members may discourage others from using certain medications, thereby reducing adherence [11].

Social support has been conceptualized as consisting of functional support, structural support, and informational support [6, 12, 13]. Functional social support includes practical help provided by an individual’s social network (e.g., providing transportation to doctor’s visits, saying encouraging words, providing care during illness) [6, 12, 13]. Structural social support refers to the number and types of connection within an individual’s social network (e.g., social network size, living arrangement, marital status) [6, 12, 13]. Informational support is the knowledge provided to an individual through their social network (e.g., providing reading material about a recent diagnosis) [6, 12, 13]. In two prior meta-analyses, functional social support was more strongly associated with treatment adherence than structural social support [6, 14]. It is unclear whether functional and structural social support affect medication adherence specifically among those with CHD risk factors other than diabetes.

In addition, how social networks operate and how social support is received may vary by race and gender. Prior studies have found that black households may have more members compared to white households to mitigate costs due to low income [12, 15, 16], blacks depend more on informal social networks for chronic disease management than whites [12, 17–19], and blacks generally have lower medication adherence compared to whites [20–24]. Moreover, differences by gender have been reported with men being more likely to report more support from their partners while women were more likely to receive support from their friends, relatives, and peers [25–27].

The aim of the present study is to investigate the associations between perceived functional and structural social support and medication adherence in a large population of black and white men and women treated with medications for CHD risk factors. Additionally, we examined whether the associations between perceived social support and medication adherence varied by race and, separately, by gender.

## Methods

### Study population

The REasons for Geographic and Racial Differences in Stroke (REGARDS) study is a cohort of 30,239 English-speaking, community-dwelling, black and white adults age 45 and older who lived in the 48 contiguous U.S. at enrollment between 2003–2007 [28]. The REGARDS study was designed to investigate racial and regional variations in stroke mortality, and oversampled black individuals and people living in the U.S. stroke buckle (coastal regions of North Carolina, South Carolina and Georgia) and the rest of the stroke belt (remaining areas of North Carolina, South Carolina and Georgia and Alabama, Arkansas, Louisiana, Mississippi, and Tennessee) [28]. The Institutional Review Boards at participating centers approved the study protocol, and all participants provided written informed consent [28].

### Data collection

Information about socio-demographic factors, cardiovascular disease risk factors, cigarette smoking, physical activity, use of medications, and psychosocial factors including perceived social support, depressive symptoms, and stress was obtained via computer assisted telephone interviews [28, 29]. Trained health professionals conducted an in-home visit to obtain systolic and diastolic blood pressure, weight and height measurements and blood and spot urine samples [29]. Fasting was requested for 10–12 hours before the in-home visit [28]. Blood and urine samples were shipped overnight with ice packs to a central laboratory at the University of Vermont and lipid profiles and glucose were obtained from laboratory assays performed on blood samples [28, 29]. Prescription and nonprescription medication use in the two weeks prior to the in-home visit was recorded by pill bottle review [28].

### Sample selection

**Fig 1:**
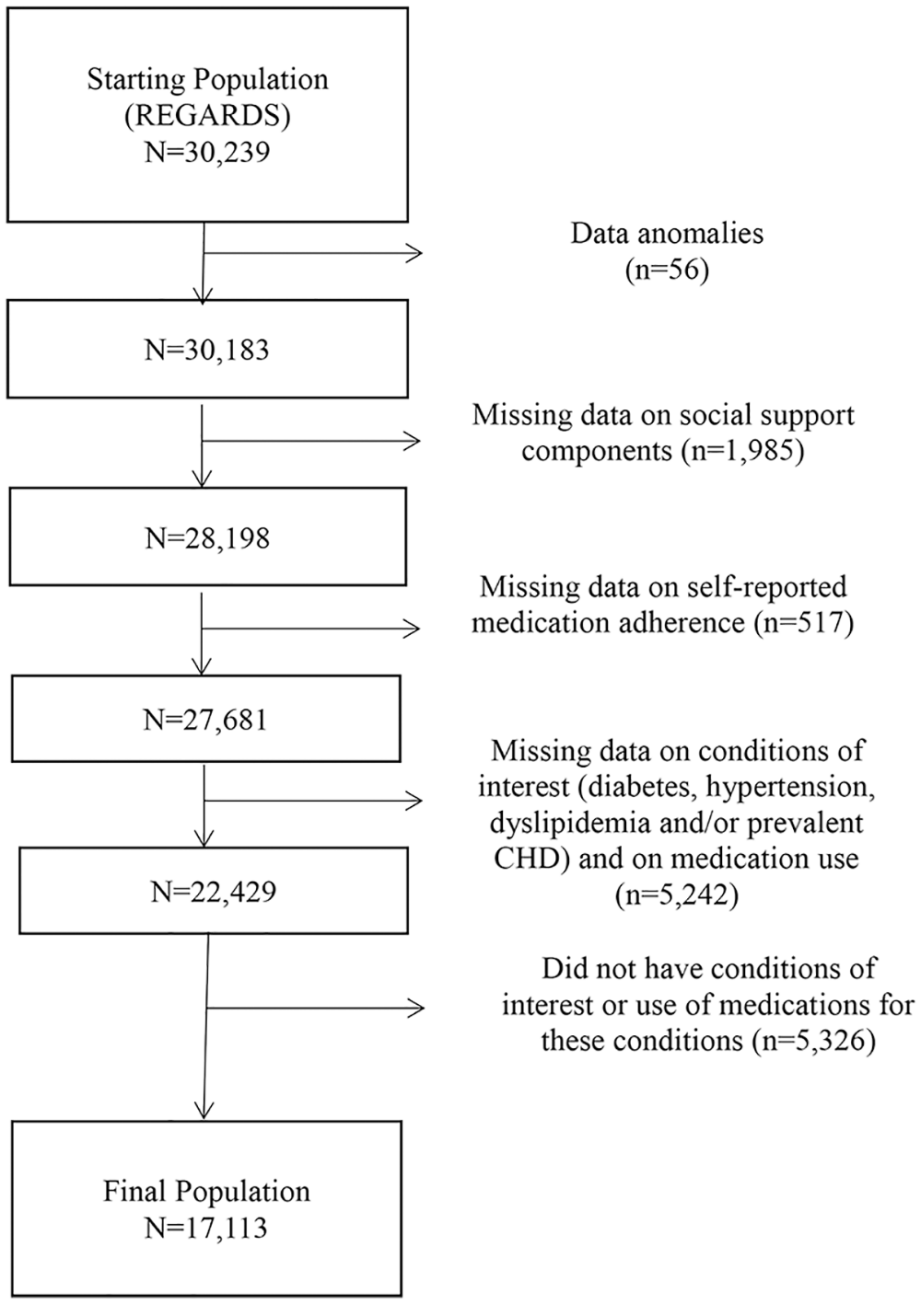
Flow chart with exclusion criteria.

For the current analyses, participants were included if they had medication-treated diabetes (use of anti-diabetes medications), hypertension (use of antihypertensive medication), or dyslipidemia (use of lipid lowering medications) and/or prevalent CHD (self-reported history or electrocardiogram [ECG] evidence of a prior myocardial infarction [MI] or self-reported coronary artery bypass graft, coronary angioplasty, or coronary stenting) and use of CHD-related medications (nitrates, nitroglycerin, clopidogrel or use of aspirin to reduce risk of MI or stroke). Participants were excluded because of data anomalies (n = 56), missing data on social support components (n = 1,985), or medication adherence (n = 517), if they were missing data on conditions of interest (diabetes, hypertension, dyslipidemia and/or prevalent CHD) or use of medications for the conditions (n = 5,242) and did not have the conditions of interest or use medications for these conditions (n = 5,326) (Fig 1). After exclusions, the sample size was 17,113 participants. Participants excluded because of missing data were more likely to be younger (64.0 years vs 66.2 years), black (41.7% vs 44.6%), have health insurance (9.1% vs 5.1%), take fewer medications (4.2 vs 7.1), have a higher mean PCS score (47.4 vs 44.7) and less likely to be female (51.5% vs 53.8%), to have prevalent CHD (18.3% vs 28.9%) and be obese (36.6% vs 44.3%) compared to those included in the study (Table A in S1 File). Participants excluded because they did not have conditions of interest or use of medications for the conditions were more likely to be younger (61.9 years vs 66.2 years), female (64.6% vs 53.8%), have health insurance (8.1% vs 5.1%), take fewer medications (3.5 vs 7.1), have a higher mean PCS score (50.4 vs 44.7) and less likely to be black (31.2% vs 44.6%), to have prevalent CHD (7.0% vs 28.9%) and to be obese (22.0% vs 44.3%) compared to those included in the study (Table A in S1 File).

### Exposures

Six survey items were used to measure perceived social support. Each social support component was considered as a separate exposure variable. Consistent with prior literature [6, 12, 13], social support was further divided into three types: functional support, structural support and a combination of functional and structural support.

#### Functional support

One item was used to measure functional support.

1. Care during illness or disability status
  Participants were asked, “If you had a serious illness or became disabled, do you have someone who would be able to provide care for you on an on going basis?” This item was dichotomized as (care during illness or disability vs no one to care during illness or disability).

#### Structural support

Four items were used to measure structural support.

1. Partnered status
  This was based on whether participants were married or in a marriage-like relationship vs divorced, widowed, separated, never been married. This was dichotomized as partnered vs not partnered respectively.
2. Number of close friends.
  Participants were asked, “How many close friends do you have? That is, people that you feel at ease with, can talk to about private matters, and can call on for help?” This was categorized based on quartiles as 0–2 close friends, 3–4 close friends, 5–6 close friends, and >6 close friends.
3. Number of close relatives
  Participants were asked, “How many relatives do you have that you feel close to?” This was categorized based on quartiles as 0–3 close relatives, 4–5 close relatives, 6–10 close relatives, and >10 close relatives.
4. Number of other adults in household
  Participants were asked, “Not counting yourself, how many adults, age 18 or older currently live in the same household with you?” Because of limited variation in this item, it was divided into tertiles as 0 other adults in household, 1 other adult in household, and >1 other adult in household.

#### Combination of functional and structural support

One item included a combination of functional and structural support.

1. Frequency of contacts
  Participants were asked “How many of these friends or relatives do you see at least once a month?” This was categorized based on quartiles as seeing 0–3 close friends or relatives at least monthly, seeing 4–5 close friends or relatives at least monthly, seeing 6–10 close friends or relatives at least monthly, and seeing >10 close friends or relatives at least monthly.

### Outcome

Medication adherence was assessed using a four-item scale (30). Participants responded yes or no to the following questions: 1) “Do you ever forget to take your medicines?”; 2) “Are you careless at times about taking your medicine?”; 3) “When you feel better, do you sometimes stop taking your medicine?” and 4) “Sometimes if you feel worse when you start taking the medicine, do you stop taking it?” The outcome was categorized as low adherence (at least one “yes” response) and high adherence (no “yes” responses), consistent with prior literature [30].

### Covariates

Access to medications results from interaction between individuals and the health system, thus the Andersen and Aday conceptual model was selected to guide the analysis. This model proposed that individual and contextual characteristics determine how and if an individual uses health services; these characteristics are categorized into predisposing, enabling, and need factors [31].

#### Pre-disposing factors

The pre-disposing factors included in the analyses were age (continuous), race (black vs. white), gender (male vs. female), region of residence (Stroke Buckle vs. Stroke Belt vs. Non-Belt), annual household income (<$20,000 vs. ≥$20,000) and education (high school graduate or less vs. some college or college graduate).

#### Enabling factors

The enabling factors available for these analyses were insurance status (yes vs. no), rural status based on Rural Urban Commuting Area [RUCA] Codes [32], (rural vs. not rural) and percentage of individuals in a zip code living below the federal poverty line (continuous).

#### Need factors

The need factors were cumulative number of medications (continuous), depressive symptoms based on the Center for Epidemiologic Studies–Depression Scale [CES-D] (CES-D score <4 vs. ≥4), physical functioning based on the Short Form 12 Physical Component Summary [PCS] score [33] (continuous), mental health based on the Short Form 12 Mental Health Component Summary (MCS) score [33] (continuous), Cohen’s perceived stress scale [34] score (continuous), general health (excellent/very good vs good vs fair/poor), obesity status based on BMI (kg/m^2^) estimated from measured height and weight during the in-home visit (obese vs not obese), physical activity (none vs. 1–3 times per week vs. ≥4 times per week) and Framingham CHD risk score: risk of coronary death or MI over 10 years among those free of CHD at baseline [35, 36] [(<10% vs 10–20% vs >20%) vs prevalent CHD].

### Statistical analysis

Participant characteristics and social support components were compared by medication adherence status (low versus high adherence) using descriptive statistics. Multivariable-adjusted Poisson regression models with robust variance estimation were used to calculate prevalence ratios (PRs) for high medication adherence for each measure of social support as follows: 1. care during illness or disability vs no one to care during illness or disability; 2. partnered vs not partnered; 3. quartiles for number of close friends with 0–2 close friends as the reference; 4. quartiles for number of close relatives with 0–3 close relatives as the reference; 5. quartiles for number of close friends or relatives seen at least monthly with seeing 0–3 close friends or relatives at least monthly as the reference, and 6. tertiles for number of other adults in household with 0 other adults in household as the reference.

First a crude model was analyzed for each exposure variable. Then, sequential adjustments were made using three models for each exposure variable based on the Andersen and Aday conceptual model [31]. The crude model was adjusted for pre-disposing factors (age, race, gender region of residence, annual household income and education) to create model 1. Model 2 was created by further adjusting model 1 for enabling factors (insurance status, rural status and percentage of individuals in a zip code living below the federal poverty line). Model 2 was further adjusted for need factors (cumulative number of medications, depressive symptoms, MCS score, perceived stress scale score, general health, obesity status, physical activity, and CHD risk category) to create model 3. Race and gender were tested separately to determine whether they were effect modifiers of the associations between high medication adherence and each of the six exposures using cross-product (interaction) terms. Multivariable-adjusted Poisson regression models with robust variance estimation as above were used to estimate PRs for high medication adherence separately for each race and gender. Multiple imputation by chained equations with ten datasets was used to account for missing covariate data [37]. The data was analyzed using SAS, version 9.4, SAS Institute, Cary, NC.

## Results

Among the 17,113 participants, the prevalence of high medication adherence was 68.9%. Participants with high medication adherence were more likely to be rural residents (20.5% vs 18.5%), to have higher mean PCS (45.0 vs 43.9) and MCS scores (54.6 vs 53.0), fewer depressive symptoms (10.3% vs 14.3%), and less perceived stress (3.0 vs 3.6) compared to participants with low medication adherence (Table 1).

**Table 1:** Characteristics of REGARDS^a^ participants by medication adherence status.

Characteristics	Medication Adherence		
	Low adherence n = 5,323	High adherence n = 11,790	p
***Predisposing factors***			
**Age, years, mean ± SD**	65.3 ± 9.0	66.7 ± 9.0	<0.0001
**Black, n (%)**	2,404 (45.2)	5,223 (44.3)	0.29
**Women, n (%)**	2,948 (55.4)	6,254 (53.0)	0.005
**Region, n (%)**			<0.0001
**Stroke belt**^b^	1,915 (36.0)	4,078 (34.6)	
**Stroke buckle**^c^	1,026 (19.3)	2,646 (22.4)	
**Non-stroke belt or buckle**	2,382 (44.8)	5,066 (43.0)	
**Annual household income <$20,000, n (%)**	1,073 (22.8)	2,294 (22.0)	0.26
**Education ≤ High school, n (%)**	2,233 (42.0)	4,809 (40.8)	
***Enabling factors***			
**No health insurance, n (%)**	285 (5.4)	585 (5.0)	0.28
**Percentage of individuals in a zip code living below the federal poverty line, mean ± SD**	17.0 ± 9.5	17.0 ± 9.3	0.92
**Rural residence, n (%)**	893 (18.5)	2,187 (20.5)	0.004
***Need factors***			
**CHD**^a^ **risk categories**^d^**, n (%)**			0.08
**< 10%**	2,271 (42.7)	5,142 (43.6)	
**10–20%**	921 (17.3)	2,132 (18.1)	
**>20%**	521 (9.8)	1,175 (10.0)	
**Prevalent CHD**^a^^e^	1,610 (30.3)	3,341 (28.3)	
**Physical activity**^f^**, n (%)**			<0.0001
**None**	2,021 (38.5)	4,212 (36.1)	
**1–3 times per week**	1,918 (36.5)	4,073 (34.9)	
**4+ times per week**	1,316 (25.0)	3,376 (29.0)	
**Cumulative number of medications ± SD**	7.2 ± 4.0	7.0 ± 3.9	0.0009
**General Health**^f^**, n (%)**			<0.0001
**Excellent/Very Good**	1,831 (34.5)	4,711 (40.0)	
**Good**	2,140 (40.3)	4,491 (38.2)	
**Fair/Poor**	1,343 (25.3)	2,565 (21.8)	
**Obesity prevalence, n (%)**	2,500 (47.4)	5,016 (42.8)	<0.0001
**Depressive symptoms, CES-D score ≥ 4, n (%)**	758 (14.3)	1,203 (10.3)	<0.0001
**Physical Component Summary Score, mean ± SD**	43.9 ± 11.0	45.0 ± 10.9	<0.0001
**Mental Component Summary Score, mean ± SD**	53.0 ± 9.1	54.6 ± 8.2	<0.0001
**Perceived Stress Scale Score, mean ± SD**	3.6 ± 3.0	3.0 ± 2.9	<0.0001

^a^Abbreviations: REGARDS, Reasons for Geographic and Racial Differences in Stroke; CHD, coronary heart disease

^b^Defined as the states of Alabama, Arkansas, Louisiana, Mississippi, Tennessee and the noncoastal regions of North Carolina, South Carolina and Georgia.

^c^Defined as the coastal regions of North Carolina, South Carolina and Georgia.

^d^Framingham CHD hard event risk score: risk of coronary death or MI over 10 years (among those free of CHD at baseline).

^e^Self-reported history or electrocardiogram (ECG) evidence of a prior myocardial infarction MI or self-reported coronary artery bypass graft, coronary angioplasty, or coronary stenting.

^f^The frequencies and percentages may not add up to the total sample size due to missing data.

In the crude analysis, participants with high medication adherence were more likely to report having someone to care for them during illness or disability (87.1% vs 84.4%), >6 close friends (24.4% vs 22.5%), >10 close relatives (16.3% vs 15.3%), to see >10 close friends or relatives at least monthly (20.5% vs 17.6%) and less likely to have >1 other adult in the household (15.4% vs 17.8%) compared to those with low medication adherence (Table 2). Compared to participants who reported seeing 0–3 close friends or relatives at least monthly, the PRs of high medication adherence for those who reported seeing 4–5 friends or relatives at least monthly, 6–10 friends or relatives at least monthly and >10 friends or relatives at least monthly were 1.03 (95% CI: 0.98, 1.09), 1.03 (95% CI: 0.99, 1.08) and 1.06 (95% CI: 1.00, 1.11) respectively, after multivariable adjustment (Table 3).

**Table 2:** Social support components by medication adherence status.

	Medication Adherence		
	Low adherence	High adherence	p
***Functional support***			
**Care during illness or disability, n (%)**	4,491 (84.4)	10,274 (87.1)	<0.0001
***Structural support***			
**Partnered, n (%)**	3,239 (60.8)	7,168 (60.8)	0.95
**Close Friends (Quartiles)**			<0.0001
**0–2 close friends, n (%)**	1,531 (28.8)	3,080 (26.1)	
**3–4 close friends, n (%)**	1,493 (28.1)	3,189 (27.1)	
**5–6 close friends, n (%)**	1,101 (20.7)	2,643 (22.4)	
**>6 close friends, n (%)**	1,198 (22.5)	2,878 (24.4)	
**Close Relatives (Quartiles)**			0.0002
**0–3 close relatives, n (%)**	2,012 (37.8)	4,057 (34.4)	
**4–5 close relatives, n (%)**	1,141 (21.4)	2,566 (21.8)	
**6–10 close relatives, n (%)**	1,357 (25.5)	3,243 (27.5)	
**>10 close relatives, n (%)**	813 (15.3)	1,924 (16.3)	
**Other adults in household (Tertiles)**			0.0004
**0 other adults in household, n (%)**	1,417 (26.6)	3,246 (27.5)	
**1 other adult in household, n (%)**	2,957 (55.6)	6,725 (57.0)	
**>1 other adult in household, n (%)**	949 (17.8)	1,819 (15.4)	
***Functional and structural support***			
**Frequency of Contacts (Quartiles)**			<0.0001
**Seeing 0–3 close friends or relativesat least monthly, n (%)**	1,957 (36.8)	3,812 (32.3)	
**Seeing 4–5 close friends or relativesat least monthly, n (%)**	1,025 (19.3)	2,320 (19.7)	
**Seeing 6–10 close friends or relativesat least monthly, n (%)**	1,402 (26.3)	3,239 (27.5)	
**Seeing >10 close friends or relativesat least monthly, n (%)**	939 (17.6)	2,419 (20.5)	

**Table 3:** Adjusted models with prevalence ratios and 95% confidence intervals of high medication adherence by social support components.

	Crude Model	Model 1^a^	Model 2^b^	Model 3^c^
	PR 95% CI	PR 95% CI	PR 95% CI	PR 95% CI
***Functional support***				
**Care during illness or disability vsNo one to care during illness or disability**	1.08 (1.02, 1.14)	1.08 (1.02, 1.14)	1.08 (1.02, 1.14)	1.05 (0.99, 1.11)
***Structural support***				
**Partnered vs not partnered**	1.00 (0.96, 1.04)	1.00 (0.96, 1.04)	1.00 (0.96, 1.04)	0.99 (0.95, 1.04)
**Close Friends (Quartiles)**				
**0–2 close friends**	Ref	Ref	Ref	Ref
**3–4 close friends**	1.02 (0.97, 1.07)	1.02 (0.97, 1.07)	1.01 (0.97, 1.07)	1.01 (0.96, 1.06)
**5–6 close friends**	1.06 (1.00, 1.11)	1.05 (1.00, 1.11)	1.05 (1.00, 1.11)	1.03 (0.98, 1.09)
**>6 close friends**	1.06 (1.00, 1.11)	1.05 (0.99, 1.10)	1.05 (0.99, 1.10)	1.02 (0.97, 1.08)
**Close Relatives (Quartiles)**				
**0–3 close relatives**	Ref	Ref	Ref	Ref
**4–5 close relatives**	1.04 (0.99, 1.09)	1.04 (0.99, 1.09)	1.04 (0.99, 1.09)	1.02 (0.97, 1.07)
**6–10 close relatives**	1.06 (1.01, 1.10)	1.05 (1.00, 1.10)	1.05 (1.00, 1.10)	1.03 (0.98, 1.08)
**>10 close relatives**	1.05 (1.00, 1.11)	1.04 (0.99, 1.10)	1.04 (0.98, 1.10)	1.02 (0.96, 1.07)
**Other adults in household (Tertiles)**				
**0 other adults in household**	Ref	Ref	Ref	Ref
**1 other adult in household**	1.00 (0.96, 1.04)	1.00 (0.96, 1.05)	1.00 (0.96, 1.05)	1.00 (0.96, 1.05)
**>1 other adult in household**	0.94 (0.89, 1.00)	0.96 (0.91, 1.02)	0.97 (0.91, 1.03)	0.97 (0.92, 1.03)
***Functional and structural support***				
**Frequency of Contacts**				
**Seeing 0–3 close friends or relativesat least monthly**	Ref	Ref	Ref	Ref
**Seeing 4–5 close friends or relativesat least monthly**	1.05 (1.00, 1.10)	1.05 (1.00, 1.10)	1.05 (1.00, 1.10)	1.03 (0.98, 1.09)
**Seeing 6–10 close friends or relativesat least monthly**	1.06 (1.01, 1.11)	1.06 (1.01, 1.11)	1.06 (1.01, 1.11)	1.03 (0.99, 1.08)
**Seeing >10 close friends or relativesat least monthly**	1.09 (1.04, 1.15)	1.09 (1.03, 1.14)	1.09 (1.03, 1.14)	1.06 (1.00, 1.11)

^a^Model 1(Pre-disposing factors): age (continuous), race (categorical), gender (categorical), region of residence (categorical), annual household income (categorical) and education (categorical).

^b^Model 2 (Enabling factors): model 1 covariates, insurance status (categorical), rural status (categorical), percentage of individuals in a zip code living below the federal poverty line (continuous).

^c^Model 3 (Need factors): model 2 covariates, cumulative number of medications (continuous), depressive symptoms, (CES-D) score (categorical), physical component summary score (continuous), mental component summary score (continuous), perceived stress scale score (continuous), general health (categorical), obesity status (categorical), physical activity (categorical), coronary heart disease risk category (categorical).

Black participants were more likely to have >1 other adult in the household compared to white participants (p <0.001) (Table B in S1 File). Women were less likely to have someone to care for them during illness or disability (p <0.001) or to be partnered (p <0.001) and were more likely to have no other adults in the household (p <0.001), compared to men (Table C in S1 File). The associations between the social support components and medication adherence were similar between groups defined by race and gender (P-values for interaction >0.10 for all exposure-effect modifier combinations) (Tables D and E in S1 File).

## Discussion

In this study of adults with CHD risk factors (diabetes, hypertension, dyslipidemia and/or prevalent CHD), the number of close friends or relatives seen at least monthly, a combination of functional and structural support, was modestly associated with higher medication adherence. The other measures of perceived social support assessed in this study, were not associated with medication adherence, once factors known to influence health services utilization were accounted for. However, overall, the prevalence of high medication adherence was notable (68.9%) given the high-risk status of this population.

Two meta-analyses indicated that functional social support had a stronger association with treatment adherence (medication adherence and adherence to other self-care activities) compared to structural social support in adults and children with a range of conditions including hypertension [6, 14]. The current study added new data which suggests that the combination of functional and structural social support via interactions with close friends or relatives may have a greater impact on medication adherence compared to other measures of functional or structural social support. Collectively, these results suggest that the quality of relationships may have a greater impact on medication adherence compared to the number of individuals in one’s social network [6]. The mechanisms behind this are unclear; it has been proposed that functional support received from relatives or friends as well as assistance provided for self-care activities facilitates medication adherence [9]. This functional support further aids individuals to cope and to be motivated and optimistic about different aspects of self-management of their chronic conditions [6, 9, 38]. As a result of supportive interactions that lead to better coping, suggested interventions to improve medication adherence include encouraging social network members to assist non-adherent members with prescription refills and pill reminders [11].

The current study may have had limited power to detect clinically important variations by race and gender in the associations between social support and medication adherence. Prior studies have found differences in the associations between social support and chronic disease self-management activities by race and gender. One study found that among women, diabetes-specific social support was associated with an increased prevalence of medication adherence among people with diabetes; however, among men, social support was not associated with medication adherence [39]. In another study, Rees and colleagues found that the association between social support and diabetes self-management activities differed by race [40]. However, medication adherence was not assessed in this study.

The strengths of the current study include the availability of data on a four-item medication adherence scale, social support components, health-related and socio-economic variables on a large population of black and white men and women from the 48 contiguous US states.

The current study has several potential limitations. This was a cross-sectional study; therefore, it was not possible to determine the temporality sequence between social support components and medication adherence. The cross-sectional nature of the study further limits our ability to make causal inferences regarding whether social support directly influences medication adherence. Since social support and medication adherence were both self-reported, it is possible that misclassification may have resulted. However, the four-item medication adherence scale used in the current analysis has been widely used, including in prior studies using the REGARDS data to evaluate anti-hypertensive medication [41] and statin [42] adherence. We relied on the participants’ perceptions of social support; we did not have information about whether unexpected support may have been provided in times of need. Further, the reporting of both social support and medication adherence may be affected by social desirability bias. Additionally, only one measure of functional support was available; therefore, this limits the ability to make further conclusions regarding the association between functional support and medication adherence. Some covariates relied also on self-report, which could have increased the potential for misclassification. Although a variety of confounders were accounted for, there was potential for residual confounding.

## Conclusions

The results of the current study indicate that among people with CHD risk factors, frequent contact with close friends or relatives (which comprises a combination of functional and structural social support) had a small association with medication adherence. Enhancing combined functional and structural social support for people with CHD risk factors such as diabetes, hypertension, dyslipidemia and prevalent CHD may help improve their medication adherence.

## Supporting information

S1 File Supporting tables. Table A. Characteristics of REGARDS participants by sample inclusion/exclusion status Table B. Social support components by race Table C. Social support components by gender Table D. Adjusted Models with prevalence ratios and 95% confidence intervals of high medication adherence by social support components among blacks and whites Table E. Adjusted Models with odds ratios and 95% confidence intervals of high medication adherence by social support components among women and men.
(DOC)

## References

[1] McDermott MM, Schmitt B, Wallner E. Impact of medication nonadherence on coronary heart disease outcomes. A critical review. Archives of Internal Medicine. 1997;157(17):1921–9. 9308504

[2] Irvine J, Baker B, Smith J, Jandciu S, Paquette M, Cairns J, et al Poor adherence to placebo or amiodarone therapy predicts mortality: results from the CAMIAT study. Canadian Amiodarone Myocardial Infarction Arrhythmia Trial. Psychosomatic Medicine. 1999;61(4):566–75. 10443767 10.1097/00006842-199907000-00023

[3] Elliott WJ, Maddy R, Toto R, Bakris G. Hypertension in patients with diabetes. Overcoming barriers to effective control. Postgraduate medicine. 2000;107(3):29–32, 5–6, 8 doi: 10.3810/pgm.2000.03.940 10728133 10.3810/pgm.2000.03.940

[4] Naderi SH, Bestwick JP, Wald DS. Adherence to drugs that prevent cardiovascular disease: meta-analysis on 376,162 patients. Am J Med. 2012;125(9):882–7. doi: 10.1016/j.amjmed.2011.12.013 22748400 10.1016/j.amjmed.2011.12.013

[5] Johnson VR, Jacobson KL, Gazmararian JA, Blake SC. Does social support help limited-literacy patients with medication adherence? A mixed methods study of patients in the Pharmacy Intervention for Limited Literacy (PILL) study. Patient Educ Couns. 2010;79(1):14–24. doi: 10.1016/j.pec.2009.07.002 19647967 10.1016/j.pec.2009.07.002

[6] DiMatteo MR. Social support and patient adherence to medical treatment: a meta-analysis. Health Psychol. 2004;23(2):207–18. doi: 10.1037/0278-6133.23.2.207 15008666 10.1037/0278-6133.23.2.207

[7] Edwards LV. Perceived social support and HIV/AIDS medication adherence among African American women. Qual Health Res. 2006;16(5):679–91. doi: 10.1177/1049732305281597 16611972 10.1177/1049732305281597

[8] Ncama BP, McInerney PA, Bhengu BR, Corless IB, Wantland DJ, Nicholas PK, et al Social support and medication adherence in HIV disease in KwaZulu-Natal, South Africa. Int J Nurs Stud. 2008;45(12):1757–63. doi: 10.1016/j.ijnurstu.2008.06.006 18653188 10.1016/j.ijnurstu.2008.06.006

[9] Gallant MP. The influence of social support on chronic illness self-management: a review and directions for research. Health Educ Behav. 2003;30(2):170–95. doi: 10.1177/1090198102251030 12693522 10.1177/1090198102251030

[10] Osborn CY, Egede LE. The relationship between depressive symptoms and medication nonadherence in type 2 diabetes: the role of social support. Gen Hosp Psychiatry. 2012;34(3):249–53. doi: 10.1016/j.genhosppsych.2012.01.015 22401705 10.1016/j.genhosppsych.2012.01.015 PMC3345067

[11] Kronish IM, Ye S. Adherence to cardiovascular medications: lessons learned and future directions. Prog Cardiovasc Dis. 2013;55(6):590–600. doi: 10.1016/j.pcad.2013.02.001 23621969 10.1016/j.pcad.2013.02.001 PMC3639439

[12] Ford ME, Tilley BC, McDonald PE. Social support among African-American adults with diabetes. Part 1: Theoretical framework. J Natl Med Assoc. 1998;90(6):361–5. 9640907 PMC2568240

[13] Uchino BN, Cacioppo JT, Kiecolt-Glaser JK. The relationship between social support and physiological processes: a review with emphasis on underlying mechanisms and implications for health. Psychological Bulletin. 1996;119(3):488–531. 8668748 10.1037/0033-2909.119.3.488

[14] Magrin ME, D'Addario M, Greco A, Miglioretti M, Sarini M, Scrignaro M, et al Social support and adherence to treatment in hypertensive patients: a meta-analysis. Ann Behav Med. 2015;49(3):307–18. doi: 10.1007/s12160-014-9663-2 25341642 10.1007/s12160-014-9663-2

[15] Wilson MN, Tolson TF. Familial support in the Black community. Journal of Clinical Child Psychology. 1990;19(4):347–55.

[16] Cohen PN, Casper LM. In Whose Home? Multigenerational Families in the United States, 1998–2000. Sociological Perspectives. 2002;45(1):1–20.

[17] Taylor RJ. Need for support and family involvement among Black Americans. Journal of Marriage and the Family. 1990:584–90.

[18] Chatters LM, Taylor RJ, Jackson JS. Size and composition of the informal helper networks of elderly blacks. J Gerontol. 1985;40(5):605–14. 3875644 10.1093/geronj/40.5.605

[19] Williams HA. A comparison of social support and social networks of black parents and white parents with chronically ill children. Social Science & Medicine. 1993;37(12):1509–20. 8303335 10.1016/0277-9536(93)90185-7

[20] Ho PM, Bryson CL, Rumsfeld JS. Medication adherence its importance in cardiovascular outcomes. Circulation. 2009;119(23):3028–35. doi: 10.1161/CIRCULATIONAHA.108.768986 19528344 10.1161/CIRCULATIONAHA.108.768986

[21] Shenolikar RA, Balkrishnan R, Camacho FT, Whitmire JT, Anderson RT. Race and medication adherence in Medicaid enrollees with type-2 diabetes. J Natl Med Assoc. 2006;98(7):1071–7. 16895275 PMC2569450

[22] Gerber BS, Cho YI, Arozullah AM, Lee SY. Racial differences in medication adherence: A cross-sectional study of Medicare enrollees. Am J Geriatr Pharmacother. 2010;8(2):136–45. doi: 10.1016/j.amjopharm.2010.03.002 20439063 10.1016/j.amjopharm.2010.03.002 PMC3740123

[23] Trinacty CM, Adams AS, Soumerai SB, Zhang F, Meigs JB, Piette JD, et al Racial differences in long-term adherence to oral antidiabetic drug therapy: a longitudinal cohort study. BMC Health Services Research. 2009;9:24 doi: 10.1186/1472-6963-9-24 19200387 10.1186/1472-6963-9-24 PMC2645384

[24] Marcum ZA, Zheng Y, Perera S, Strotmeyer E, Newman AB, Simonsick EM, et al Prevalence and correlates of self-reported medication non-adherence among older adults with coronary heart disease, diabetes mellitus, and/or hypertension. Research in Social & AdministrativePpharmacy: RSAP. 2013;9(6):817–27. 23291338 10.1016/j.sapharm.2012.12.002 PMC3620923

[25] Kaplan RM, Hartwell SL. Differential effects of social support and social network on physiological and social outcomes in men and women with type II diabetes mellitus. Health Psychol. 1987;6(5):387–98. 3678167 10.1037//0278-6133.6.5.387

[26] Enzlin P, Mathieu C, Demyttenaere K. Gender differences in the psychological adjustment to type 1 diabetes mellitus: an explorative study. Patient Educ Couns. 2002;48(2):139–45. 12401417 10.1016/s0738-3991(02)00009-5

[27] Tang TS, Brown MB, Funnell MM, Anderson RM. Social support, quality of life, and self-care behaviors amongAfrican Americans with type 2 diabetes. The Diabetes Educator. 2008;34(2):266–76. doi: 10.1177/0145721708315680 18375776 10.1177/0145721708315680

[28] Howard VJ, Cushman M, Pulley L, Gomez CR, Go RC, Prineas RJ, et al The reasons for geographic and racial differences in stroke study: objectives and design. Neuroepidemiology. 2005;25(3):135–43. doi: 10.1159/000086678 15990444 10.1159/000086678

[29] Gutierrez OM, Khodneva YA, Muntner P, Rizk DV, McClellan WM, Cushman M, et al Association between urinary albumin excretion and coronary heart disease in black vs white adults. JAMA. 2013;310(7):706–14. doi: 10.1001/jama.2013.8777 23989654 10.1001/jama.2013.8777 PMC3837520

[30] Morisky DE, Green LW, Levine DM. Concurrent and predictive validity of a self-reported measure of medication adherence. Med Care. 1986;24(1):67–74. 3945130 10.1097/00005650-198601000-00007

[31] Aday LA, Andersen R. A Framework for the Study of Access to Medical Care. Health Services Research. 1974;9(3):208–20. 4436074 PMC1071804

[32] Larson SL, Fleishman JA. Rural-urban differences in usual source of care and ambulatory service use: analyses of national data using Urban Influence Codes. Medical Care. 2003;41(7):III-65–III-74. 12865728 10.1097/01.MLR.0000076053.28108.F2

[33] Quality Metric. The SF-12s: an even shorter health survey. www.sf-36.org/tools/sf12.shtml. 2007.

[34] Cohen S, Kamarck T, Mermelstein R. A global measure of perceived stress. J Health Soc Behav. 1983;24(4):385–96. 6668417

[35] D'Agostino RB Sr., Grundy S, Sullivan LM, Wilson P. Validation of the Framingham coronary heart disease prediction scores: results of a multiple ethnic groups investigation. JAMA. 2001;286(2):180–7. 11448281 10.1001/jama.286.2.180

[36] Wilson PW, D'Agostino RB, Levy D, Belanger AM, Silbershatz H, Kannel WB. Prediction of coronary heart disease using risk factor categories. Circulation. 1998;97(18):1837–47. 9603539 10.1161/01.cir.97.18.1837

[37] Royston P, White IR. Multiple Imputation by Chained Equations (MICE): Implementation in Stata. 2011 2011;45(4):20.

[38] Albus C. Psychological and social factors in coronary heart disease. Annals of Medicine. 2010;42(7):487–94. doi: 10.3109/07853890.2010.515605 20839918 10.3109/07853890.2010.515605

[39] Mondesir FL, White K, Liese AD, McLain AC. Gender, Illness-Related Diabetes Social Support, and Glycemic Control Among Middle-Aged and Older Adults. J Gerontol B Psychol Sci Soc Sci. 2016;71(6):1081–8. doi: 10.1093/geronb/gbv061 26307487 10.1093/geronb/gbv061

[40] Rees CA, Karter AJ, Young BA. Race/ethnicity, social support, and associations with diabetes self-care and clinical outcomes in NHANES. The Diabetes Educator. 2010;36(3):435–45. doi: 10.1177/0145721710364419 20332281 10.1177/0145721710364419 PMC2878375

[41] Irvin MR, Shimbo D, Mann DM, Reynolds K, Krousel-Wood M, Limdi NA, et al Prevalence and correlates of low medication adherence in apparent treatment-resistant hypertension. Journal of Clinical Hypertension (Greenwich, Conn). 2012;14(10):694–700. 10.1111/j.1751-7176.2012.00690.x PMC3464920 23031147

[42] Glasser SP, Wadley V, Judd S, Kana B, Prince V, Jenny N, et al The association of statin use and statin type and cognitive performance: analysis of the reasons for geographic and racial differences in stroke (REGARDS) study. Clinical Cardiology. 2010;33(5):280–8. doi: 10.1002/clc.20758 20513066 10.1002/clc.20758 PMC2925406

