# Supplementary material for: Association of functional and structural social support with medication adherence among individuals treated for coronary heart disease risk factors: Findings from the REasons for Geographic and Racial Differences in Stroke (REGARDS) study
Source: PLoS One. 2018 Jun 27;13(6):e0198578. doi: 10.1371/journal.pone.0198578 (PMC6021050; doi:10.1371/journal.pone.0198578)
Supplement: S1 File — Table A. Characteristics of REGARDS participants by sample inclusion/exclusion status Table B. Social support components by race Table C. Social support components by gender Table D. Adjusted Models with prevalence ratios and 95% confidence intervals of high medication adherence by social support components among blacks and whites Table E. Adjusted Models with odds ratios and 95% confidence intervals of high medication adherence by social support components among women and men. (DOC) [file pone.0198578.s001.doc]

**Table A. Characteristics of REGARDS^a^ participants by sample inclusion/exclusion status**

|  | **Sample** | | | |
| --- | --- | --- | --- | --- |
| **Characteristics** | **Included** | **Excluded** | | **p** |
|  |  | **Missing data** | **No conditions of interest** |  |
|  | n = 17,113 | n = 7,744 | n = 5,326 |  |
| ***Predisposing factors*** |  |  |  |  |
| **Age, years, mean ± SD** | 66.2 ± 9.1 | 64.0 (9.5) | 61.6 (9.6) | <0.0001 |
| **Black, n (%)** | 7,627 (44.6) | 3,226 (41.7) | 1,661 (31.2) | <0.0001 |
| **Women, n (%)** | 9,202 (53.8) | 3,990 (51.5) | 3,440 (64.6) | <0.0001 |
| **Region, n (%)** |  |  |  | 0.0004 |
| **Stroke belt^b^** | 5,993 (35.0) | 2,666 (34.4) | 1,788 (33.6) |  |
| **Stroke buckle^c^** | 3,672 (21.5) | 1,590 (20.5) | 1,045 (19.6) |  |
| **Non-stroke belt or buckle** | 7,448 (43.5) | 3,488 (45.0) | 2,493 (46.8) |  |
| **Annual household income <$20,000, n (%)** | 3,367 (22.2) | 1,437 (21.6) | 674 (14.5) | <0.0001 |
| **Education ≤ High school, n (%)** | 7,042 (41.2) | 3,056 (39.5) | 1,498 (28.1) | <0.0001 |
| ***Enabling factors*** |  |  |  |  |
| **No health insurance, n (%)** | 870 (5.1) | 705 (9.1) | 430 (8.1) | <0.0001 |
| **Percentage of individuals in a zip code**  **living below the federal poverty line, mean ± SD** | 17.0 ± 9.3 | 17.1 (9.4) | 15.0 (9.2) | <0.0001 |
| **Rural residence, n (%)** | 3,080 (19.9) | 1,423 (20.3) | 862 (18.0) | 0.006 |
| ***Need factors*** |  |  |  |  |
| **CHD^a^ risk categories^d^, n (%)** |  |  |  | <0.0001 |
| **< 10%** | 7,413 (43.3) | 3,542 (45.7) | 4,353 (81.8) |  |
| **10-20%** | 3,053 (17.8) | 1,773 (22.9) | 507 (9.5) |  |
| **>20%** | 1,696 (9.9) | 1,011 (13.1) | 90 (1.7) |  |
| **Prevalent CHD^ae^** | 4,951 (28.9) | 1,418 (18.3) | 374 (7.0) |  |
| **Physical activity^f^, n (%)** |  |  |  | <0.0001 |
| **None** | 6,233 (36.9) | 2,541 (33.6) | 1,466 (28.0) |  |
| **1-3 times per week** | 5,991 (35.4) | 2,703 (35.7) | 2,001 (38.1) |  |
| **4+ times per week** | 4,692 (27.7) | 2,327 (30.7) | 1,779 (33.9) |  |
| **Cumulative number of medications ± SD** | 7.1 ± 4.0 | 4.2 (3.8) | 3.5 (3.5) | <0.0001 |
| **General Health^f^, n (%)** |  |  |  | <0.0001 |
| **Excellent/Very Good** | 6,542 (38.3) | 3,809 (49.4) | 3,620 (68.0) |  |
| **Good** | 6,631 (38.8) | 2,633 (34.1) | 1,311 (24.6) |  |
| **Fair/Poor** | 3,908 (22.9) | 1,275 (16.5) | 391 (7.4) |  |
| **Obesity prevalence, n (%)** | 7,516 (44.3) | 2,815 (36.6) | 1,168 (22.0) | <0.0001 |
| **Depressive symptoms, CES-D score ≥ 4, n (%)** | 1,961 (11.5) | 910 (11.8) | 478 (9.1) | <0.0001 |
| **Physical Component Summary Score, mean ± SD** | 44.7 ± 10.9 | 47.4 (10.2) | 50.4 (8.6) | <0.0001 |
| **Mental Component Summary Score, mean ± SD** | 54.1 ± 8.5 | 53.7 (8.7) | 54.2 (7.9) | 0.0007 |
| **Perceived Stress Scale Score, mean ± SD** | 3.2 ± 3.0 | 3.3 (3.0) | 3.1 (2.7) | 0.0005 |

^a^Abbreviations: REGARDS, Reasons for Geographic and Racial Differences in Stroke; CHD, coronary heart

disease

^b^Defined as the states of Alabama, Arkansas, Louisiana, Mississippi, Tennessee and the noncoastal regions of

North Carolina, South Carolina and Georgia.

^c^Defined as the coastal regions of North Carolina, South Carolina and Georgia

^d^Framingham CHD hard event risk score: risk of coronary death or MI over 10 years (among those free of CHD at baseline)

^e^Self-reported history or electrocardiogram (ECG) evidence of a prior myocardial infarction MI or self-reported coronary

artery bypass graft, coronary angioplasty, or coronary stenting

^f^The frequencies and percentages may not add up to the total sample size due to missing data.

**Table B. Social support components by race**

|  | **Race** | |  |
| --- | --- | --- | --- |
|  | **Blacks**  **n = 7,627** | **Whites**  **n=9,486** | **p** |
| ***Functional support*** |  |  |  |
| **Care during illness or disability, n (%)** | 6,554 (85.9) | 8,211 (86.6) | 0.24 |
| ***Structural support*** |  |  |  |
| **Partnered, n (%)** | 3,664 (48.0) | 6,743 (71.1) | <0.0001 |
| **Close Friends (Quartiles)** |  |  | <0.0001 |
| **0-2 close friends, n (%)** | 2,628 (34.5) | 1,983 (20.9) |  |
| **3-4 close friends, n (%)** | 2,303 (30.2) | 2,379 (25.1) |  |
| **5-6 close friends, n (%)** | 1,406 (18.4) | 2,338 (24.7) |  |
| **>6 close friends, n (%)** | 1,290 (16.9) | 2,786 (29.4) |  |
| **Close Relatives (Quartiles)** |  |  | 0.05 |
| **0-3 close relatives, n (%)** | 2,777 (36.4) | 3,292 (34.7) |  |
| **4-5 close relatives, n (%)** | 1,643 (21.5) | 2,064 (21.8) |  |
| **6-10 close relatives, n (%)** | 2,040 (26.8) | 2,560 (27.0) |  |
| **>10 close relatives, n (%)** | 1,167 (15.3) | 1,570 (16.6) |  |
| **Other adults in household (Tertiles)** |  |  | <0.0001 |
| **0 other adults in household, n (%)** | 2,346 (30.8) | 2,317 (24.4) |  |
| **1 other adult in household, n (%)** | 3,651 (47.9) | 6,031 (63.6) |  |
| **>1 other adult in household, n (%)** | 1,630 (21.4) | 1,138 (12.0) |  |
| ***Functional and structural support*** |  |  |  |
| **Frequency of Contacts** |  |  | <0.0001 |
| **Seeing 0-3 close friends or relatives**  **at least monthly, n (%)** | 2,848 (37.3) | 2,921 (30.8) |  |
| **Seeing 4-5 close friends or relatives**  **at least monthly, n (%)** | 1,525 (20.0) | 1,820 (19.2) |  |
| **Seeing 6-10 close friends or relatives**  **at least monthly, n (%)** | 2,007 (26.3) | 2,634 (27.8) |  |
| **Seeing >10 close friends or relatives**  **at least monthly, n (%)** | 1,247 (16.4) | 2,111 (22.3) |  |

**Table C. Social support components by gender**

|  | **Gender** | |  |
| --- | --- | --- | --- |
|  | **Women**  **n=9,202** | **Men**  **n=7,911** | **p** |
| ***Functional support*** |  |  |  |
| **Care during illness or disability, n (%)** | 7,634 (83.0) | 7,131 (90.1) | <0.0001 |
| ***Structural support*** |  |  |  |
| **Partnered, n (%)** | 4,044 (44.0) | 6,363 (80.4) | <0.0001 |
| **Close Friends (Quartiles)** |  |  | <0.0001 |
| **0-2 close friends, n (%)** | 2,625 (28.5) | 1,986 (25.1) |  |
| **3-4 close friends, n (%)** | 2,793 (30.4) | 1,889 (23.9) |  |
| **5-6 close friends, n (%)** | 2,007 (21.8) | 1,737 (22.0) |  |
| **>6 close friends, n (%)** | 1,777 (19.3) | 2,299 (29.1) |  |
| **Close Relatives (Quartiles)** |  |  | <0.0001 |
| **0-3 close relatives, n (%)** | 3,457 (37.6) | 2,612 (33.0) |  |
| **4-5 close relatives, n (%)** | 2,098 (22.8) | 1,609 (20.3) |  |
| **6-10 close relatives, n (%)** | 2,381 (25.9) | 2,219 (28.1) |  |
| **>10 close relatives, n (%)** | 1,266 (13.8) | 1,471 (18.6) |  |
| **Other adults in household (Tertiles)** |  |  | <0.0001 |
| **0 other adults in household, n (%)** | 3,388 (36.8) | 1,275 (16.1) |  |
| **1 other adult in household, n (%)** | 4,378 (47.6) | 5,304 (67.1) |  |
| **>1 other adult in household, n (%)** | 1,436 (15.6) | 1,332 (16.8) |  |
| ***Functional and structural support*** |  |  |  |
| **Frequency of Contacts (Quartiles)** |  |  | <0.0001 |
| **Seeing 0-3 close friends or relatives**  **at least monthly, n (%)** | 3,104 (33.7) | 2,665 (33.7) |  |
| **Seeing 4-5 close friends or relatives**  **at least monthly, n (%)** | 1,845 (20.1) | 1,500 (19.0) |  |
| **Seeing 6-10 close friends or relatives**  **at least monthly, n (%)** | 2,573 (28.0) | 2,068 (26.1) |  |
| **Seeing >10 close friends or relatives**  **at least monthly, n (%)** | 1,680 (18.3) | 1,678 (21.2) |  |

**Table D. Adjusted Models with prevalence ratios and 95% confidence intervals of high medication adherence by social support components among blacks and whites**

|  | **Blacks** | | | | **Whites** | | | |  |
| --- | --- | --- | --- | --- | --- | --- | --- | --- | --- |
|  | **Crude**  **Model** | **Model 1^a^** | **Model 2^b^** | **Model 3^c^** | **Crude Model** | **Model 1^a^** | **Model 2^b^** | **Model 3^c^** |  |
|  | **PR**  **95% CI** | **PR**  **95% CI** | **PR**  **95% CI** | **PR**  **95% CI** | **PR**  **95% CI** | **PR**  **95% CI** | **PR**  **95% CI** | **PR**  **95% CI** | **p^d^** |
| ***Functional support*** | | |  |  |  |  |  |  |  |
| **Care during illness or**  **disability**  **vs none** | 1.11  (1.02, 1.20) | 1.11  (1.02, 1.20) | 1.11  (1.02, 1.20) | 1.07  (0.99, 1.17) | 1.05  (0.98, 1.13) | 1.06  (0.98, 1.14) | 1.06  (0.98, 1.14) | 1.03  (0.96, 1.11) | 0.36 |
| ***Structural support*** | | | | | | | | | |
| **Partnered vs not partnered** | 0.99  (0.94, 1.05) | 0.98  (0.92, 1.04) | 0.98  (0.92, 1.04) | 0.98  (0.92, 1.04) | 1.00  (0.95, 1.06) | 1.02  (0.96, 1.09) | 1.02  (0.96, 1.08) | 1.01  (0.95, 1.08) | 0.74 |
| **Close Friends (Quartiles)** | | | | | | | | | 0.39 |
| **0-2 close friends** | Ref | Ref | Ref | Ref | Ref | Ref | Ref | Ref |  |
| **3-4 close friends** | 1.01  (0.94, 1.08) | 1.00  (0.94, 1.08) | 1.00  (0.94, 1.08) | 1.01  (0.96, 1.06) | 1.04  (0.96, 1.11) | 1.04  (0.96, 1.11) | 1.04  (0.96, 1.11) | 1.02  (0.95, 1.10) |  |
| **5-6 close friends** | 1.03  (0.95, 1.11) | 1.02  (0.94, 1.10) | 1.02  (0.94, 1.10) | 1.03  (0.98, 1.09) | 1.08  (1.01, 1.16) | 1.08  (1.01, 1.16) | 1.08  (1.01, 1.16) | 1.06  (0.98, 1.14) |  |
| **>6 close**  **friends** | 1.03  (0.95, 1.12) | 1.01  (0.93, 1.10) | 1.01  (0.94, 1.10) | 1.02  (0.96, 1.07) | 1.08  (1.01, 1.16) | 1.08  (1.00, 1.15) | 1.08  (1.00, 1.15) | 1.05  (0.97, 1.12) |  |
| **Close Relatives (Quartiles)** | | | | | | | | | 0.74 |
| **0-3 close relatives** | Ref | Ref | Ref | Ref | Ref | Ref | Ref | Ref |  |
| **4-5 close relatives** | 1.02  (0.95, 1.11) | 1.02  (0.95, 1.10) | 1.02  (0.95, 1.10) | 1.01  (0.94, 1.09) | 1.05  (0.98, 1.12) | 1.05  (0.98, 1.12) | 1.05  (0.98, 1.12) | 1.03  (0.97, 1.10) |  |
| **6-10 close relatives** | 1.03  (0.96, 1.11) | 1.02  (0.96, 1.10) | 1.02  (0.96, 1.10) | 1.01  (0.94, 1.08) | 1.04  (0.97, 1.12) | 1.07  (1.01, 1.14) | 1.07  (1.00, 1.14) | 1.05  (0.99, 1.12) |  |
| **>10 close relatives** | 1.06  (0.98, 1.15) | 1.05  (0.97, 1.14) | 1.05  (0.97, 1.14) | 1.02  (0.94, 1.11) | 1.07  (1.01, 1.14) | 1.03  (0.96, 1.11) | 1.03  (0.96, 1.11) | 1.01  (0.94, 1.09) |  |
| **Other adults in household (Tertiles)** | | | | | | | | | 0.50 |
| **0 other adults in household** | Ref | Ref | Ref | Ref | Ref | Ref | Ref | Ref |  |
| **1 other adult in household** | 0.99  (0.93, 1.05) | 0.99  (0.92, 1.05) | 1.00  (0.92, 1.05) | 1.00  (0.95, 1.04) | 1.00  (0.95, 1.06) | 1.02  (0.95, 1.08) | 1.02  (0.95, 1.08) | 1.01  (0.95, 1.08) |  |
| **>1 other adult in household** | 0.97  (0.90, 1.05) | 0.98  (0.91, 1.06) | 0.97  (0.91, 1.03) | 0.97  (0.92, 1.03) | 0.91  (0.83, 0.99) | 0.94  (0.86, 1.03) | 0.94  (0.86, 1.03) | 0.95  (0.86, 1.04) |  |
| ***Functional and structural support*** | | | | | | | | | |
| **Frequency of Contacts (Quartiles)** | | | | | | | | | 0.52 |
| **Seeing 0-3 close friends or relatives at least monthly** | Ref | Ref | Ref | Ref | Ref | Ref | Ref | Ref |  |
| **Seeing 4-5 close friends or relatives at least monthly** | 1.03  (0.95, 1.11) | 1.03  (0.96, 1.11) | 1.03  (0.96, 1.11) | 1.02  (0.94, 1.10) | 1.07  (0.99, 1.15) | 1.06  (0.99, 1.14) | 1.06  (0.99, 1.14) | 1.05  (0.98, 1.12) |  |
| **Seeing 6-10 close friends or relatives at least monthly** | 1.07  (0.99, 1.14) | 1.07  (0.99, 1.14) | 1.07  (0.99, 1.14) | 1.04  (0.97, 1.12) | 1.05  (0.98, 1.12) | 1.05  (0.98, 1.12) | 1.05  (0.98, 1.12) | 1.03  (0.96, 1.10) |  |
| **Seeing >10 close friends or relatives at least monthly** | 1.07  (0.99, 1.16) | 1.07  (0.98, 1.15) | 1.07  (0.98, 1.16) | 1.03  (0.95, 1.12) | 1.10  (1.03, 1.18) | 1.10  (1.03, 1.18) | 1.10  (1.03, 1.18) | 1.07  (1.00, 1.15) |  |

^a^Model 1 (Pre-disposing factors): age (continuous), gender (categorical), region of residence (categorical), annual household income (categorical) and education (categorical).

^b^Model 2 (Enabling factors): model 1 covariates, insurance status (categorical), rural status (categorical), percentage of individuals in a zip code living below the federal poverty line (continuous).

^c^Model 3 (Need factors): model 2 covariates, cumulative number of medications (continuous), depressive symptoms, (CES-D) score (categorical), physical component summary score (continuous), mental component summary score (continuous), perceived stress scale score (continuous), general health (categorical), obesity status (categorical), physical activity (categorical), coronary heart disease risk category (categorical).

^d^P-value for interaction (Model 3)

**Table E. Adjusted Models with odds ratios and 95% confidence intervals of high medication adherence by social support components among women and men**

|  | **Women** | | | | **Men** | | | |  |
| --- | --- | --- | --- | --- | --- | --- | --- | --- | --- |
|  | **Crude**  **Model** | **Model 1^a^** | **Model 2^b^** | **Model 3^c^** | **Crude Model** | **Model 1^a^** | **Model 2^b^** | **Model 3^c^** |  |
|  | **PR**  **95% CI** | **PR**  **95% CI** | **PR**  **95% CI** | **PR 95% CI** | **PR**  **95% CI** | **PR 95% CI** | **PR 95% CI** | **PR 95% CI** | **p^d^** |
| ***Functional support*** | | | | | | | | | |
| **Care during**  **illness or**  **disability**  **vs none** | 1.09  (1.02, 1.16) | 1.09  (1.02, 1.17) | 1.09  (1.02, 1.17) | 1.07  (1.00, 1.15) | 1.05  (0.96, 1.15) | 1.05  (0.96, 1.15) | 1.05  (0.96, 1.15) | 1.01  (0.92, 1.11) | 0.51 |
| ***Structural support*** | | | | | | | | | |
| **Partnered**  **vs not**  **partnered** | 0.99  (0.94, 1.04) | 1.00  (0.95, 1.06) | 1.00  (0.95, 1.06) | 1.01  (0.95, 1.08) | 0.99  (0.92, 1.06) | 1.00  (0.93, 1.07) | 0.99  (0.93, 1.06) | 0.98  (0.91, 1.05) | 0.71 |
| **Close Friends (Quartiles)** | | | | | | | | | 0.79 |
| **0-2**  **close friends** | Ref | Ref | Ref | Ref | Ref | Ref | Ref | Ref |  |
| **3-4**  **close friends** | 1.03  (0.97, 1.11) | 1.04  (0.96, 1.11) | 1.04  (0.96, 1.11) | 1.02 (0.95,1.09) | 1.00  (0.93, 1.08) | 1.00  (0.93, 1.08) | 1.00  (0.93, 1.08) | 0.99  (0.91, 1.07) |  |
| **5-6**  **close friends** | 1.06  (0.99, 1.13) | 1.08  (1.01, 1.16) | 1.08  (1.01, 1.16) | 1.03  (0.96, 1.11) | 1.05  (0.98, 1.14) | 1.06  (0.98, 1.14) | 1.06  (0.98, 1.14) | 1.03  (0.96, 1.12) |  |
| **>6 close**  **friends** | 1.06  (0.99, 1.14) | 1.08  (1.00, 1.15) | 1.08  (1.00, 1.15) | 1.03  (0.95, 1.11) | 1.04  (0.97, 1.12) | 1.04  (0.97, 1.12) | 1.04  (0.97, 1.12) | 1.01  (0.94, 1.09) |  |
| **Close Relatives (Quartiles)** | | | | | | | | | 0.99 |
| **0-3 close**  **relatives** | Ref | Ref | Ref | Ref | Ref | Ref | Ref | Ref |  |
| **4-5 close**  **relatives** | 1.05  (0.98, 1.12) | 1.05  (0.98, 1.12) | 1.05  (0.98, 1.12) | 1.04  (0.97, 1.11) | 1.02  (0.95, 1.10) | 1.02  (0.95, 1.10) | 1.02  (0.95, 1.10) | 1.00  (0.93, 1.08) |  |
| **6-10 close**  **relatives** | 1.04  (0.98, 1.11) | 1.03  (0.97, 1.10) | 1.03  (0.97, 1.10) | 1.02  (0.95, 1.08) | 1.07  (1.00, 1.14) | 1.06  (1.00, 1.14) | 1.06  (0.99, 1.14) | 1.05  (0.98, 1.12) |  |
| **>10 close**  **relatives** | 1.05  (0.98, 1.14) | 1.04  (0.97, 1.13) | 1.04  (0.97, 1.13) | 1.02  (0.94, 1.10) | 1.04  (0.97, 1.13) | 1.04  (0.96, 1.12) | 1.04  (0.96, 1.12) | 1.01  (0.94, 1.09) |  |
| **Other adults in household (Tertiles)** | | | | | | | | | 0.83 |
| **0 other**  **adults in**  **household** | Ref | Ref | Ref | Ref | Ref | Ref | Ref | Ref |  |
| **1 other adult**  **in household** | 0.98  (0.93, 1.04) | 1.00  (0.94, 1.06) | 1.00  (0.94, 1.06) | 1.00  (0.95, 1.06) | 1.00  (0.93, 1.08) | 1.01  (0.94, 1.09) | 1.01  (0.93, 1.09) | 0.99  (0.92, 1.07) |  |
| **>1 other**  **adult in**  **household** | 0.95  (0.88, 1.02) | 0.98  (0.91, 1.06) | 0.98  (0.91, 1.06) | 0.99  (0.92, 1.07) | 0.93  (0.85, 1.02) | 0.95  (0.86, 1.04) | 0.95  (0.86, 1.04) | 0.95  (0.86, 1.04) |  |
| ***Functional and structural support*** | | | | | | | | | |
| **Frequency of Contacts (Quartiles)** | | | | | | | | | 0.54 |
| **Seeing 0-3 close friends or relatives at least monthly** | Ref | Ref | Ref | Ref | Ref | Ref | Ref | Ref |  |
| **Seeing 4-5**  **close friends**  **or relatives**  **at least**  **monthly** | 1.03  (0.96, 1.10) | 1.02  (0.95, 1.10) | 1.02  (0.95, 1.10) | 1.01  (0.94, 1.08) | 1.08  (1.00, 1.16) | 1.08  (1.00, 1.16) | 1.08  (1.00, 1.16) | 1.06  (0.99, 1.15) |  |
| **Seeing 6-10**  **close friends**  **or relative at least monthly** | 1.06  (0.99, 1.13) | 1.05  (0.99, 1.12) | 1.05  (0.99, 1.12) | 1.03  (0.97, 1.10) | 1.06  (0.98, 1.13) | 1.06  (0.99, 1.13) | 1.06  (0.99, 1.13) | 1.04  (0.97, 1.11) |  |
| **Seeing >10**  **close friends**  **or relatives**  **at least**  **monthly** | 1.12  (1.04, 1.20) | 1.11  (1.03, 1.19) | 1.11  (1.03, 1.19) | 1.08  (1.00, 1.16) | 1.06  (0.99, 1.14) | 1.07  (0.99, 1.15) | 1.07  (0.99, 1.15) | 1.04  (0.96, 1.12) |  |

^a^Model 1 (Pre-disposing factors): age (continuous), race (categorical), region of residence (categorical), annual household income (categorical) and education (categorical).

^b^Model 2 (Enabling factors): model 1 covariates, insurance status (categorical), rural status (categorical), percentage of individuals in a zip code living below the federal poverty line (continuous).

^c^Model 3 (Need factors): model 2 covariates, cumulative number of medications (continuous), depressive symptoms, (CES-D) score (categorical), physical component summary score (continuous), mental component summary score (continuous), perceived stress scale score (continuous), general health (categorical), obesity status (categorical), physical activity (categorical), coronary heart disease risk category (categorical).

^d^P-value for interaction (Model 3)
